# Accelerating multiscale modelling of fluids with on-the-fly Gaussian process regression

**DOI:** 10.1007/s10404-018-2164-z

**Published:** 2018-11-16

**Authors:** David Stephenson, James R. Kermode, Duncan A. Lockerby

**Affiliations:** 1 School of Engineering, University of Warwick, Coventry, CV4 7AL UK; 2 Warwick Centre for Predictive Modelling, School of Engineering, University of Warwick, Coventry, CV4 7AL UK

**Keywords:** Multiscale modelling, Machine learning, Hybrid methods, Micro/nanofluidics, Molecular dynamics

## Abstract

We present a scheme for accelerating hybrid continuum-atomistic models in multiscale fluidic systems by using Gaussian process regression as a surrogate model for computationally expensive molecular dynamics simulations. Using Gaussian process regression, we are able to accurately predict atomic-scale information purely by consideration of the macroscopic continuum-model inputs and outputs and judge on the fly whether the uncertainty of our prediction is at an acceptable level, else a new molecular simulation is performed to continually augment the database, which is never required to be complete. This provides a substantial improvement over the current generation of hybrid methods, which often require many similar atomistic simulations to be performed, discarding information after it is used once. We apply our hybrid scheme to nano-confined unsteady flow through a high-aspect-ratio converging–diverging channel, and make comparisons between the new scheme and full molecular dynamics simulations for a range of uncertainty thresholds and initial databases. For low thresholds, our hybrid solution is highly accurate—around that of thermal noise. As the uncertainty threshold is raised, the accuracy of our scheme decreases and the computational speed-up increases (relative to a full molecular simulation), enabling the compromise between accuracy and efficiency to be tuned. The speed-up of our hybrid solution ranges from an order of magnitude, with no initial database, to cases where an extensive initial database ensures no new MD simulations are required.

## Introduction

Almost all fluid engineering systems are multiscale in their nature. At the smallest scale, the fluid and surrounding environment are comprised of atoms, with interactions occurring across nanometers ($10^{-9}$ m) and over femtoseconds ($10^{-15}$ s), while the fluid flow is characterized by the scale of the system geometry, which is often many orders of magnitude larger. In most instances, the separation of scales is so large that the atomistic behaviour can be accurately incorporated into a continuum fluid description through empirical boundary conditions (e.g. the no-slip condition at walls) and constitutive relations (e.g. viscosity in the shear stress–strain rate relation). However, as some characteristic dimension of the system approaches the micro/nanoscale, these approximations break down, and the fluid flow becomes highly dependent on atomistic phenomena (Schoch et al. 2008; Hadjiconstantinou 1999; Brenner et al. 1994; Karniadakis et al. 2005).

A major challenge in modern computational fluid dynamics is how to capture these microscopic effects without incurring a prohibitive simulation cost. There are numerous applications where atomistic information is required to capture non-continuum/non-equilibrium phenomena, but the macroscopic flow develops over much larger length and time scales; e.g. pumping technology that exploits thermal creep in a rarefied gas (Patronis and Lockerby 2014), or high-throughput nanotube membranes for salt water desalination (Ritos et al. 2015). The multiscale nature of these systems leads to a dual requirement for capturing the local atomic-scale interactions and macro-scale fluid response. The complexity of the flow necessitates modelling with atomic resolution, but the state-of-the-art techniques (molecular dynamics (MD) for dense fluid flows (Allen and Tildesley 1987), and the direct simulation Monte-Carlo method (DSMC) for rarefied gas flows (Bird 1994) are extremely computationally expensive. This limits their application to small system sizes, typically ${\mathcal {O}}(100\hbox { nm}^3$), and short simulation times, typically ${\mathcal {O}}(100\hbox { ns})$, rendering many important engineering problems intractable, and limiting possibilities for comparison with experiments.

Hybrid methods provide a promising framework for simulating such systems by combining continuum (macro) and atomistic (micro) solvers and exploiting scale separation where it exists to obtain a highly accurate, yet computationally tractable, solution. Hybrid approaches to fluid dynamics problems are a well-researched area (e.g. see recent reviews by Wijesinghe and Hadjiconstantinou (2004), Hadjiconstantinou (2005), Kalweit and Drikakis (2008), and Mohamed and Mohamad (2009). Broadly speaking, hybrid methods operate by identifying the regions which require a micro-resolution, then coupling the micro- and macro-domains together via the exchange of state or flux variables to ensure consistency; the information passed from one model is used as a boundary condition for the other. The majority of hybrid methods provide a concurrent approach to multiscale modelling (Delgado-Buscalioni et al. 2008; Markesteijn et al. 2017), i.e. both the micro- and macro-simulations are performed at the same time. A common criticism of concurrent hybrid methods is that they require the repetitive simulation of similar micro-configurations—i.e. information from the micro-domain is used once then wastefully discarded, before regenerating similar information in a future simulation.

An alternative, sequential, hybrid approach is to use look-up tables, whereby micro-simulations are performed ahead of time with the information stored in a table (Walter et al. 2013; Holland et al. 2015; Borg and Reese 2017). This table is then used as a surrogate model for all micro-simulations, with the macro-model interpolating between data entries whenever it requires micro-input. The drawback of such a scheme is that either a) the micro-simulations do not cover a sufficiently wide range of parameter values (or are too sparsely spread), leading to poor interpolation/extrapolation accuracy; or b) the micro-simulations cover too wide a range of parameter values (or are overly numerous) and information from many of the simulations are not used. In this paper, we propose a hybrid method which uses a surrogate model to replace costly micro-simulations, but can judge on-the-fly when the surrogate’s prediction is likely to be poor. At this point, a new micro-simulation can be automatically performed and added to a growing database which never needs to be complete. In this way, we combine the best aspects of concurrent and sequential approaches. The aim is to optimize the information efficiency of the most computationally expensive part of a hybrid method by reducing the number of superfluous micro-simulations—to do this, we use machine learning.

Machine learning (ML) is a popular umbrella term for a wide variety of inferential data-driven methods. In recent years, machine learning techniques have been employed to cheaply incorporate nanoscale information into more coarse-grained models, e.g. building quantum-mechanics-informed molecular force fields sequentially (Behler and Parrinello 2007; Bartók et al. 2010; Szlachta et al. 2014; Botu et al. 2017) and on the fly (Li et al. 2015; Botu and Ramprasad 2015; Caccin et al. 2015); predicting atomisation energies of organic molecules from density-functional theory (Rupp et al. 2012); and informing continuum stress calculations with molecular dynamics (Ulz and Moran 2012). Machine learning has also been used to aid hybrid methods in fluid dynamics: to quantify the uncertainty propagating from the micro- to the macro-model as a function of time-averaging window and the amount of sampled data (Salloum et al. 2012); constructing a constitutive relation for a continuum model that is applicable to nanoscale physics (Salloum and Templeton 2014); and building a surrogate model to replace MD simulations using neural networks (NNs) (Asproulis and Drikakis 2013) and Gaussian processes (GPs) (Salloum and Templeton 2014). However, all such approaches, bar that in Asproulis and Drikakis (2013) are limited by the training data used to fit the ML model; i.e. they are sequential hybrid methods, and are not transferable to situations not envisaged at the time of construction.

In Asproulis and Drikakis (2013), an NN is used for the surrogate model; here, we choose to use GP regression which has the advantages of a) directly capturing the model uncertainty by outputting probability distributions for the predicted values—this provides a simple measure for prediction accuracy; and b) being simple to design, with models described by only a few parameters which can be easily optimised. This produces a natural trade-off between fitting the data and smoothing, and this well-tuned smoothing has the added benefit of permitting micro-simulation times to be kept short.

The goal of this paper is to demonstrate the first-ever on-the-fly implementation of GP regression into a hybrid fluidic model, providing an accessible introduction to the confluence of two fields: machine learning and multiscale fluid dynamics. The paper is laid out as follows: first, we introduce the benchmark fluidic system and the relevant multiscale method, we then explain how the GP regression is performed and implemented, and finally we present results and discussion.

## Methodology

**Fig. 1:**
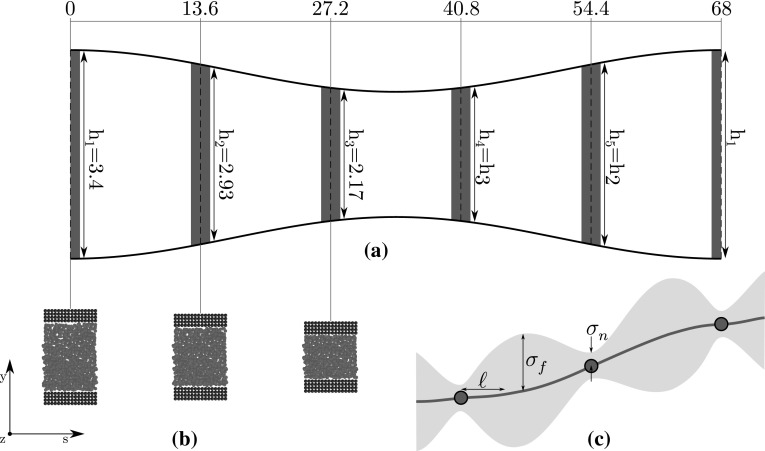
Schematics of **a** the multiscaled converging–diverging nanochannel, **b** the micro-subdomain decomposition, and **c** a 1D representation of the Gaussian-process surrogate model, where the points are observed measurements from the micro-model, the blue line is the surrogate prediction (the mean of the posterior distribution), and the grey envelope is a confidence threshold for the prediction (based on the variance of the posterior distribution). All dimensions are in nm

The system we will use as a benchmark for our new scheme is dense fluid flow through a converging–diverging channel, with the flow driven by a time-variant periodic external force $F_{\mathrm {ext}}(t)$. The geometry of the system is presented in Fig. 1a. We choose this as our benchmark system for two reasons: 1) it is multiscale both spatially and temporally; and 2) the results for the full atomistic simulation, recently published by Borg et al. (2015), provide a useful basis for comparison. The hybrid method we use to model this system is the unsteady Internal-flow Multiscale Method (IMM), developed by Borg et al. (2015), and described in detail therein. For completeness, we provide a short summary below.

### Hybrid method

Our benchmark system has a high aspect ratio, with non-continuum effects (e.g. velocity slip and density layering) persisting over the entire cross section; as such, spatial scale separation can only be exploited in the streamwise direction. Micro-subdomain simulations cover the entire channel height and are placed at regular intervals in the streamwise *s*-direction. The channel is periodic in the *s*-direction, so the first micro-subdomain is simultaneously located at the inlet and the outlet. The number of micro-subdomains *N* is set large enough to resolve the streamwise geometrical variation; here we, like Borg et al., use $N=5$. The channel height *h*(*s*) varies sinusoidally with streamwise position from 3.4 nm at the inlet/outlet to 2.04 nm at the centre. Each micro-simulation is considered to be in quasi-steady state because the characteristic time for the evolution of the macro-model (e.g. the period of the external force) is much larger than the characteristic time for the development of the micro-model (e.g. the start-up time from rest).

The macro-model consists of the unsteady one-dimensional equations for mass and momentum conservation. We use MD for the micro-model, with atoms interacting through pairwise potentials and moving according to Newton’s laws of motion (see Appendix A for details). Coupling is performed by ensuring that the mass and momentum in each micro-subdomain are consistent with the conservation laws of the macro-model. For mass:

1 $$\begin{aligned} \frac{\partial \rho }{\partial t} + \left( \frac{1}{A}\right) \frac{\partial q}{\partial s} = 0, \end{aligned}$$

where $\rho (s,t)$ is the density, *A* is the cross-sectional area, and *q*(*s*,*t*) is the time-averaged mass flow rate. Micro-subdomains are also perdiodic in the *s*-direction and cannot support a pressure gradient; therefore, for the momentum flux to be hydrodynamically equivalent to that in the macro-model, the total force *F*(*s*,*t*) applied to each atom is

2 $$\begin{aligned} F = F_{\mathrm {ext}} - \left( \frac{m}{\rho }\right) \frac{\mathrm {d}p}{\mathrm {d}s}, \end{aligned}$$

where *m* is the mass of a single atom.

### Gaussian process regression

In this paper, we replace the majority of micro-simulations with a cheaper data-driven surrogate model to negate much of the computational cost. The challenge here is to produce a relationship between the macroscopic inputs (channel height *h*, density $\rho$, and force *F*) and the microscopic output (predominantly mass flow rate *q*), despite no prior knowledge of the function form (other than it is smooth), and to determine, on the fly, when this relationship is likely to be inaccurate. For this, we use GP regression. Here, we provide only a brief overview of the approach; see Rasmussen and Williams (2006) for further details. In a GP, the predicted output at every point $\mathbf{x }$ in some continuous multi-dimensional input space is modelled by a normally distributed random variable^1^, i.e. across all of input space, our unknown relationship $q=f(\mathbf{x })$ is described entirely by a probability distribution with a mean function $\mu$ and a covariance function *C*. Here, our input space is 3-dimensional, so the $i^{\mathrm {th}}$ input $\mathbf{x }_i = (h_i,\rho _i,F_i)$.

Our surrogate model learns through gathering data via micro-simulations, but we must start from some prior belief of what our function looks like, i.e. an initial estimate of the probability distribution for mass flow rate, before data is considered. The posterior belief in the function, after the data has been taken into account, is calculated by Bayesian inference. The mean of the posterior distribution is the prediction of the surrogate model—it is the expected value of mass flow rate given the observed data and our prior belief—and the variance of the posterior distribution is a measure of the uncertainty of the prediction. The prediction will closely resemble the measured mass flow rates $\mathbf{q }$ near the input data points *X* with a high degree of confidence—i.e. low posterior variance. However, away from the observed data the prediction will approximate the prior mean function with low confidence—i.e. high posterior variance (see Fig. 1c for an illustration). Predictions, thus, become more accurate as the database grows and covers more of input space. As each data point ‘speaks’, the GP can be considered to have a finite, but unbounded, number of parameters, which grow with the database.

For mathematical simplicity, we choose the prior mean function to be

3 $$\begin{aligned} \mu (\mathbf{x }) = 0. \end{aligned}$$

A covariance function models the correlation between predictions $f (\textbf{x}_i)$ and $f (\textbf{x}_j)$ at inputs $\mathbf{x }_i$ and $\mathbf{x }_j$, respectively. In this paper, we use the squared exponential kernel *K* for the prior covariance function because it is stationary (it is only dependent on the relative position of inputs, not their absolute values) and is simple (it is only comprised of two hyperparameters):

4 $$\begin{aligned} K(\mathbf{x }_i,\mathbf{x }_j) = \sigma _f^2 \exp \left( -\frac{d_{ij}^2}{2\ell ^2}\right) , \end{aligned}$$

where $\sigma _f^2$ and $\ell$ are the two hyperparameters, representing the signal variance and the length scale of the unknown function, respectively (see Fig. 1c). The signal variance is a scaling factor defining the variance of the predictions away from known data; the length scale describes the function smoothness and the spearation distance between inputs before their respective predictions become uncorrelated. The term $d_{ij}^2$ is the squared Euclidean distance between the points $\mathbf{x }_i$ and $\mathbf{x }_j$ in input space, normalized by the mean separation for each input variable—this allows a single length scale to be used for simplicity (see Appendix B for details).

We assume that the observed mass flow rates differ from the function values by some additive noise (because the micro-simulation measurements are not perfectly accurate), i.e. $q = f(\mathbf{x }) + \epsilon$ where the noise $\epsilon$ is normally distributed with a mean of zero and a variance of $\sigma _n^2$ 2. This noise variance is an additional hyperparameter. The prior covariance between the mass flow rate observations is then

5 $$\begin{aligned} \mathrm {cov}(\mathbf{q }) = C(X,X) = K(X,X) + \sigma _n^2I, \end{aligned}$$

where *I* is the identity matrix. For a set of test inputs $X_*$, Bayesian inference leads to a posterior distribution for mass flow rate predictions $\mathbf{q }_*$, with a mean

6 $$\begin{aligned} \bar{\mathbf{f }}_* = \mu (X_*) + K(X_*,X) C(X,X)^{-1}\left( \mathbf{q } - \mu (X) \right) , \end{aligned}$$

and a covariance

7 $$\begin{aligned} \mathrm {cov}({\mathbf {f}}_*) = K(X_*,X_*) - K(X_*,X) C(X,X)^{-1}K(X,X_*). \end{aligned}$$

The prediction variances $\sigma _{f_*}^2$ are the diagonal of the posterior covariance matrix $\mathrm {cov}({\mathbf {f}}_*)$. In our scheme, whenever the standard deviation of the mass flow rate prediction $\sigma _{f_*}$ exceeds a pre-determined uncertainty threshold $\sigma _t$, the prediction is deemed insufficiently accurate and a new micro-simulation is automatically performed and added to the database.

The values of the hyperparameters are important to ensure that we do not over- or under-fit to the data. While the noise variance can be calculated directly from instantaneous mass flow rate observations in a training set of micro-simulations ($\sigma _n = 0.05$ ng/s), the remaining two hyperparameters must be numerically optimised using maximum likelihood estimation (MLE) over the same training data. In MLE, hyperparameters are chosen such that the resulting function is most consistent with the observed mass flow rates. For the training data, we used a small sample which would later be used as an initial database for case D4 (see Table 1 in Sect. 3.1). Performing MLE yielded results of $\sigma _f \approx 1$ ng/s and $\ell \approx 1$, which were both rounded to unity for simplicity. We also applied MLE to covariance model selection, comparing the squared-exponential kernel,the Mateŕn 3/2 kernel, and Mateŕn 5/2 kernel, with negligible difference found between them (maximum likelihoods within 3% of each other).

### Implementation

The step-by-step procedure for implementing our GP-accelerated hybrid method is now described, with reference to the variables in our benchmark system. In this section, subscripts denote a position index (i.e. different subdomains) and superscripts denote a (macroscale) time index.

1. Generate a range of data points for each microscale input variable $X=(h, \varvec{\rho }, F)$ and perform the requisite micro-simulations. Calculate the time-averaged mean output variable(s) to be passed to the macro-model ${\bar{q}}$.
2. If possible, measure the variance of the output variable *q* directly from the simulations. This is the noise variance hyperparameter $\sigma _n$.
3. Use MLE over the training data (see Rasmussen and Williams 2006 for more details) to set the remaining hyperparameters $\sigma _f$ and $\ell$ (and $\sigma _n$ if it could not be set in the prior step). Compare the MLE across different kernels to ensure a sensible model has been chosen.
4. Use Eqs. (4) and (5) to calculate the covariance between the training set outputs.
5. Repeat steps 2, 3, and 4 for each output variable. For our benchmark system, we used a separate 2-input GP as an equation of state to calculate pressure *p* from density and channel height. This GP was trained over hundreds of inputs using a single simulation, for each different micro-subdomain height, at negligible computational cost. As this database was very easy to cheaply fill, it was not updated on the fly. The hyperparameters for this GP were $\sigma _n=0.003$ MPa, and $\sigma _f$ and $\ell$ were again calculated to be approximately one.
6. Choose an initial database with which to start the hybrid simulation. This can be empty if desired.
7. Set the hybrid simulation parameters and initial conditions. For our benchmark system, this is the number of micro-subdomains *N*, the distance between micro-subdomains *S*, the height of each micro-subdomain *h* (see Fig. 1), the macroscopic runtime and time-step, the initial density distribution $\varvec{\rho }_{1...N}^1=\left\{ 1331, 1320, 1278, 1273, 1312\right\}$ $\hbox {kg/m}^3$, the external forcing function $F_{\mathrm {ext}}$, and the uncertainty threshold $\sigma _t$.
8. Evolve the macro-model in time.
9. Calculate the pressure using GP regression. To do this, use Eq. (4) to calculate the similarity between the current macro-state $X_* = (\mathbf{h }_{1...N},\varvec{\rho }_{1...N}^i)$ and the training inputs $X_p$. Hence calculate the pressure across all micro-subdomains using Eq. (6).
10. Calculate the pressure gradient at each micro-subdomain along the channel using a central difference approximation.
11. Calculate the total force $\mathbf{F }_{1...N}^i$ to applied to each atom, for each micro-subdomain.
12. Microscale information is now required, so query the surrogate GP for each micro-subdomain in turn. Use Eq. (4) to calculate the similarity between the current macro-state $\mathbf{x }_* = (h_j, \rho ^i_j, F^i_j)$ and the training inputs $X_q$. Hence calculate the mass flow rate prediction ${\bar{f}}_*$ using Eq. (6) and the uncertainty of said prediction $\sigma _{f_*}$ using Eq. (7). Note, as we are making a prediction for a single point in input space, the outputs of Eqs. (6) and (7) are both scalars, rather than a vector and a matrix, respectively.
13. If $\sigma _{f_*}> \sigma _t$, then a new micro-simulation is performed with input $\mathbf{x }_*$; otherwise ${\bar{f}}_*$ is taken as the mass flow rate output ${\bar{q}}^i_j$ of the micro-model.
14. If a new micro-simulation is performed, append the initial database with the input $\mathbf{x }_* = (h_j, \rho ^i_j, F^i_j)$ and the time-averaged output ${\bar{q}}^i_j$. Append the covariance matrix with this new data point $C(\left[ X\; \mathbf{x }_*\right] ,\left[ X\; \mathbf{x }_*\right] )$ using Eqs. (4) and (5).
15. Once steps 12, 13, and 14 have been completed for each micro-subdomain, the density distribution across the channel can be calculated using a finite-difference form of Eq. (2).
16. Repeat steps 8 onwards until the macro simulation is complete.

For the micro-model, to minimize the start-up time before measurements can be made, simulations are initiated with the final atomistic positions and velocities of the nearest configuration from our database ($x_{\mathrm {in}}$), i.e. the configuration with which it has the highest covariance $K(x_{\mathrm {in}},x_*)$. If there is no database entry for the channel height to be tested, new MD simulations start with the fluid atoms in a simple cubic lattice, with zero mean velocity. We estimate the start-up time for each MD simulation by performing a unique ‘pseudo MD’ simulation (at trivial computational cost) using a 1D Navier–Stokes solver with a Navier slip condition.

## Results and discussion

Since experimental results are not available for this system, we test the accuracy of our hybrid scheme by comparing it to a full MD simulation of the same system; this also enables us to directly quantify the computational savings of our scheme. All the full MD solutions presented here are taken from Borg et al. (2015), with data points representing block averages over 2000 time-steps to reduce noise. For the majority of the results we present, the external forcing $F_{\mathrm {ext}}$ is sinusoidal with an amplitude of $F_A=0.487$ pN and a period of $T=10.8$ ns (Case C in Borg et al. 2015), i.e.

8 $$\begin{aligned} F_{\mathrm {ext}}(t) = F_A\sin \left( \frac{2\pi t}{T}\right) . \end{aligned}$$

Let us first consider the most computationally demanding case, where we start with an empty MD database. With no human intuition to prescribe a likely useful set of starting data, all learning must occur on-the-fly with our GP regression surrogate model. While it is fairly straightforward to estimate the input ranges for our benchmark system (as explained later), this may not be true for more complex systems with a larger number of input variables; it is, therefore, important to demonstrate that our scheme is sufficiently robust to accurately model the flow behaviour with no prior information. Nevertheless, setting the target uncertainty threshold $\sigma _t$ involves some subjectivity. A sensible approach is to set the threshold above the measurement noise ($\sigma _n$), because it is difficult for the model to make predictions with more accuracy than the data upon which it is based^3^. We initially choose a threshold of $\sigma _t=0.1$ ng/s, twice that of $\sigma _n$. This will be referred to as Case 1.

**Fig. 2:**
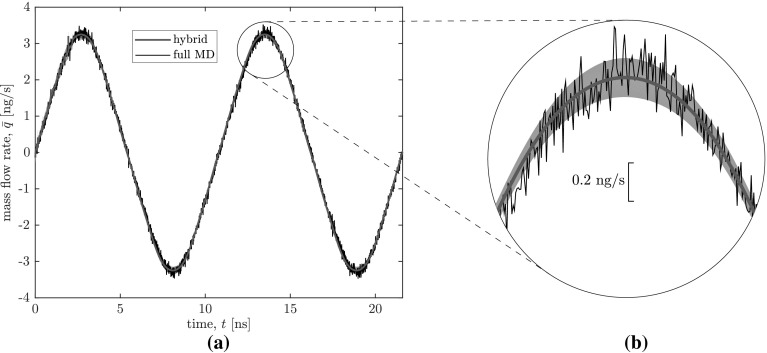
Transient mass flow rate results for our hybrid scheme (blue line; Case 1—micro-subdomain #1) and the full MD simulation (black line; Borg et al. 2015), showing **a** the full time series, and **b** a close-up to highlight the uncertainty of our hybrid solution—the grey envelope is drawn 1.96 standard deviations above and below of the mean, representing the 95% confidence interval. The hybrid solution uses a tight uncertainty threshold of 0.1 ng/s and starts from an empty database

The transient mass flow rate results for Case 1 are displayed in Fig. 2, showing excellent agreement between the output of our hybrid scheme and the measurements from the full MD simulation. Mass conservation means that the mass flow rate profile is approximately the same at all micro-subdomain locations, so we present the data only for micro-subdomain 1. As we begin from an empty database, initially our hybrid scheme must perform micro-simulations with high frequency, because there is limited data upon which to base a prediction. Therefore, the hybrid solution (blue line) exhibits noise similar to that of the full MD simulation up until $t=2.7$ ns, where the external forcing function peaks. As the system geometry and external forcing function are both symmetric, after this time no ‘new’ input configurations are encountered, and no further micro-simulations need to be performed. Beyond this time, our hybrid solution near-perfectly captures the sinusoidal temporal variation of mass flow rate, with smoothness resulting from our choice of covariance function.

Figure 2b shows a close-up of the second mass flow rate peak and highlights that the uncertainty of our hybrid solution (grey region, representing 95% confidence bounds) is smaller than the noise in the full MD simulation. This is to be expected because our micro-simulations are performed in the steady state; in the full MD simulation, properties are transient while mass flow rate are time-averaged. The uncertainty of our surrogate model’s mass flow rate prediction is larger at the extremes because these configurations exhibit the most extreme force and density inputs, and thus the model is extrapolating beyond its existing database.

### Uncertainty threshold and initial database size

**Fig. 3:**
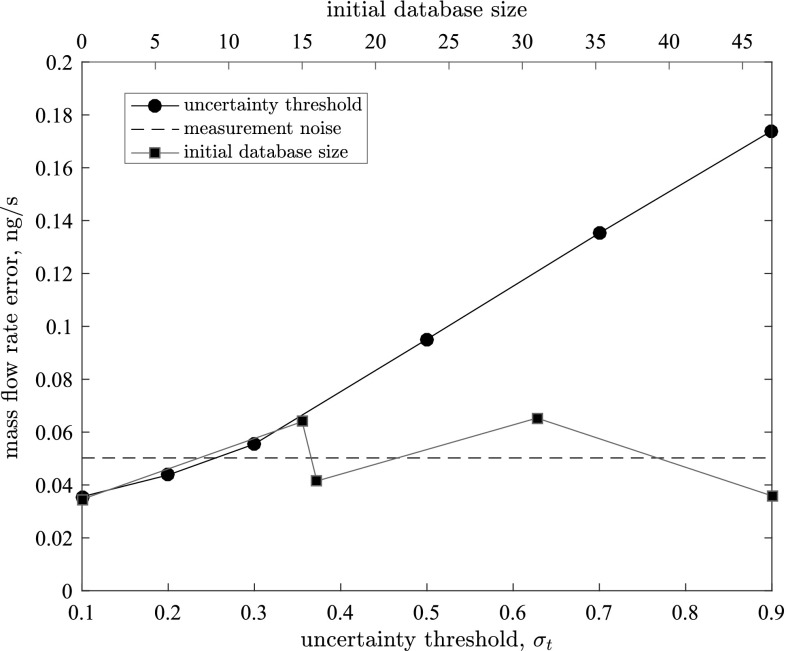
The influence of the uncertainty threshold $\sigma _t$ (starting from an empty database) and initial database size (for $\sigma _t=0.1$ ng/s) on the accuracy of our hybrid solution for mass flow rate. The horizontal dashed line represents the measurement noise for our surrogate model

Cases T1–T5 and D1–D4 demonstrate the effect of the uncertainty threshold and the initial database size on our hybrid solution. To isolate the effect of the uncertainty threshold, Cases T1–T5 all start with an empty database while the threshold varies from 0.2 ng/s (case T1) to 0.9 ng/s (case T5). Similarly, to isolate the effect of the initial database size, the uncertainty threshold is kept at a constant 0.1 ng/s for cases D1–D4, while the database varies from 15 micro-cimulations (case D1) to 47 (case D4). The construction of each initial database is outlined in Table 1. Due to symmetry, the channel height for every micro-simulation will be either $h_1$, $h_2$, or $h_3$ (see Fig. 1) and each initial database has learned from micro-simulations of specific channel heights, as listed in Table 1. For each channel height, four densities and four forces are learned, uniformly distributed across an estimated useful range^4^. For the force, this ranges from zero to the magnitude of the external force; for the density, this ranges from $1120, \hbox {kg/m}^3$ to $1480\, \hbox {kg/m}^3$.

**Table 1:** Initial databases for Cases D1–D4. See Fig. 1 for channel height references

Case	Channel heights	Initial database size
D1	$h_1$	15
D2	$h_3$	16
D3	$h_1, h_3$	31
D4	$h_1, h_2, h_3$	47

Figure 3 confirms that a larger uncertainty threshold for our surrogate model yields greater error for the hybrid solution, while the initial database size has a negligible effect. As the threshold is raised, micro-simulations are performed less frequently, so the accuracy of our hybrid solution drops. The signal standard deviation is $\sigma _f=1$ ng/s (see Sect. 2.2), so when the threshold $\sigma _t>1$ ng/s, micro-simulations will never be performed, even when starting from an empty database. In this instance, the mass flow rate in each micro-subdomain is predicted to be zero for the entire time series, as this is the prior mean. The mass flow rate error is the discrepancy between our hybrid solution and full MD simulation, averaged over all micro-subdomains and all macro time-steps for each case:

9 $$\begin{aligned} \mathrm {error} = \sum _{i=1}^{N_t}\sum _{j=1}^N({\bar{q}}_j^i - q_{f_i}), \end{aligned}$$

where $N_t = t_{\mathrm {end}}/\Delta t$ is the number of macro time-steps and $q_{f_i}$ is the mass flow rate in the full MD simulation. To obtain a smooth error, the noise from the full MD solution is filtered by performing GP regression over the raw data, using a periodic kernel with time as the single input variable.^5^ Fig. 3 shows that the error remains in the region of the measurement noise up to $\sigma _t=0.3$ ng/s, after which it increases dramatically up to three times the measurement noise when $\sigma _t=0.9$ ng/s. Varying the initial database has a negligible effect on the mass flow rate error; this is expected because the larger uncertainty encountered by having to extrapolate more often from a small database is countered by learning more frequently on-the-fly.

**Fig. 4:**
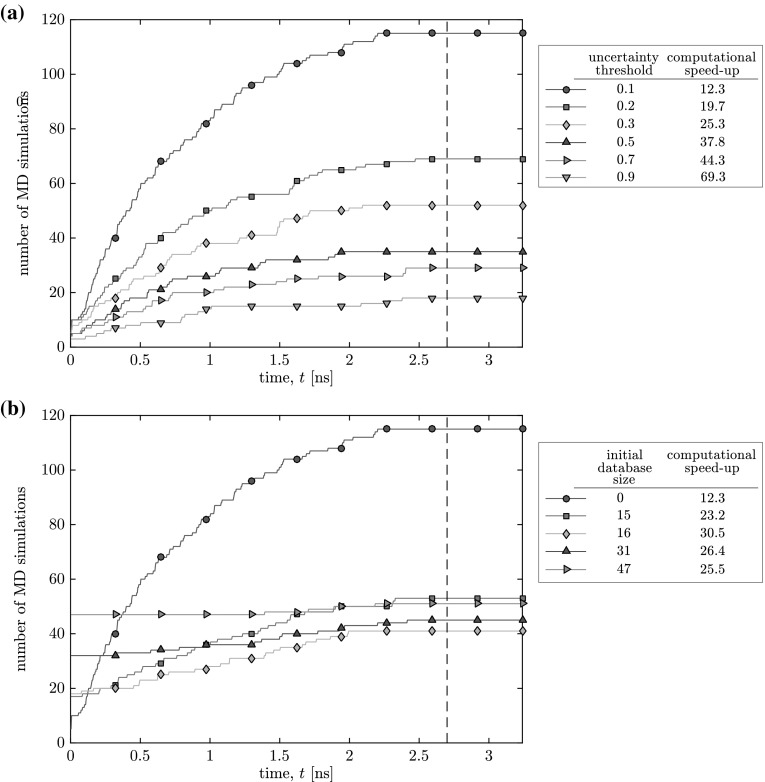
The influence of **a** the uncertainty threshold $\sigma _t$ (starting from an empty database, Cases 1 and T1–T5) and **b** initial database size (for $\sigma _t=0.1$ ng/s, Cases 1 and D1–D4) on the computational efficiency of our hybrid solution for mass flow rate. The vertical dashed line denotes time for the first peak in the external forcing function, after which no further micro-simulations are performed

Figure 4 demonstrates how the computational speed of our scheme varies with the uncertainty threshold and initial database size by measuring the cumulative number of micro-simulations. As expected, the trend is that the lower the threshold, the more micro-simulations must be performed. In all cases, no further micro-simulations are required after $t=2.7$ ns. The computational speed-up over the full MD simulation is calculated by

10 $$\begin{aligned} \mathrm {speed\text{-}up} = \frac{t_{\mathrm {end}}N_{a_f}}{{\bar{t}}_{\mathrm {sim}}N_{\mathrm {sim}}{\bar{N}}_{a_h}}, \end{aligned}$$

where $N_{a_f}$ is the number of atoms in the full MD simulation, ${\bar{t}}_{\mathrm {sim}}$ is the average time-steps performed in a single micro-subdomain simulation, $N_{\mathrm {sim}}$ is the number of micro-subdomain simulations performed for the hybrid solution, and ${\bar{N}}_{a_h}$ is the average number of atoms in each of those micro-simulations. For the tightest threshold (Case 1), our hybrid solution provides a modest speed-up over the full MD simulation of $12.3\times$; this rises to $69.3\times$ for the loosest threshold (Case T5), confirming our intuition that the choice of uncertainty threshold is a trade off between accuracy and computational efficiency. All cases show logarithmic growth for the number of required micro-simulations with respect to time— i.e. the frequency of MD simulations decreases as the database becomes larger, and the predictions become more accurate.

The total number of micro-simulations decreases when the initial database is not empty, because more predictions are made through interpolation and ‘new’ configurations are not encountered so frequently at the start of the time series. However, the total number of micro-simulations performed does not continue to fall as the initial database grows. For larger initial databases, redundant information is sometimes gathered and never encountered in the dynamical simulation. For example, consider the discrepancy between the results of Cases D1 and D2, despite the model learning only one extra configuration for the latter case. This is due to the geometry of the case: a much larger force is required near the throat of the channel (micro-subdomains with $h_3$) than at the inlet/outlet (micro-subdomains with $h_1$) to generate equal mass flow rates.^6^ As such, the local pressure gradient always acts in the opposite direction to the external force at the inlet/outlet of the channel and the peak force applied to micro-simulations is relatively small; thus learning the mass flow rate response for large forces in a channel of height $h_1$ provides little information. Conversely, all of the information is used when the initial database is formed using channel heights of $h_3$. Fewer micro-simulations corresponds to an increase in computational efficiency—Case D2 is $30.5\times$ faster than the full MD simulation while maintaining a high level of accuracy (error of 0.042 ng/s). Another example is the difference between the results for Cases D3 and D4, where including micro-simulations with channel height $h_2$ only negligibly reduces the number of ‘on-the-fly’ simulations performed because much of this information can be inferred from simulations of other channel heights.

### Building on an existing database

As we have already demonstrated, one important advantage of using GP regression is that it enables information to be stored in and reused from a continually-growing database. So far, this information has been reused within the same case, resulting in decreasing the uncertainty of our mass flow rate predictions for configurations encountered later in the time series. However, we can go further. For example, suppose having completed the hybrid simulation, we decided that we are really interested in a flow feature occurring at $s=6.8$ nm (halfway between micro-subdomains #1 and #2). Our previous options would have been to run the expensive full MD simulation to ensure every flow feature is captured, or to perform a new hybrid simulation using different micro-subdomain locations; both of which are computationally wasteful. However, using GP regression, we can simply create a new case which has micro-subdomains more frequently located, with the surrogate model having already learned from the database that we generated in the previous case.

**Fig. 5:**
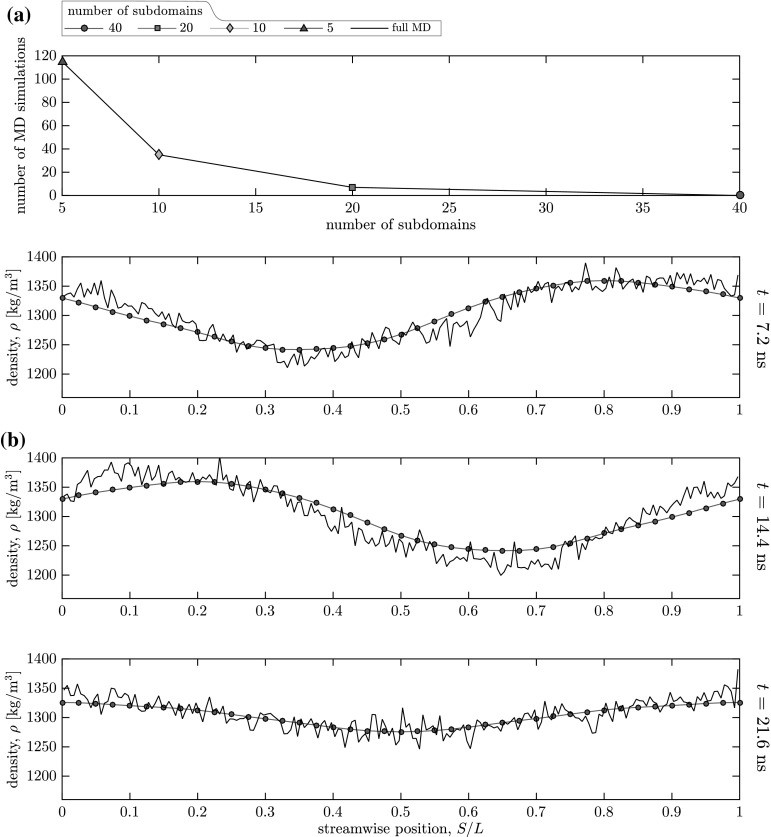
The influence of the number of micro-subdomains on the hybrid solution for density (with an uncertainty threshold of 0.1 ng/s): **a** the number of new micro-simulations (Cases 1 ans S1–S3); and **b** streamwise density profiles for the full MD simulation and the hybrid solution for Case S3 using 40 micro-subdomains at $t = 7.2$ ns, $t=14.4$ ns, and $t=21.6$ ns

In Cases S1–S3, we demonstrate how this approach can be used to continually add micro-subdomains and refine the streamwise density profile. In each case, the number of micro-subdomains is doubled (with the new micro-subdomain locations bisecting the old micro-subdomain locations), starting from $N=10$—double that of Case 1. The total database generated at the end of the previous case is used as the initial database for the subsequent case. For all cases, the uncertainty threshold is $\sigma _t=0.1$ ng/s. As the spacing between adjacent micro-subdomains decreases, the relevance of data measured at neighbouring micro-subdomains increases, and successively fewer micro-simulations are performed, as shown in Fig. 5a. Using 40 micro-subdomains, no new micro-simulations are required at all during the dynamical simulation. In addition, the accuracy of streamwise density profiles increases with the number of micro-subdomains. This is because central differences are used to model spatial gradients in the macro-simulation, which assumes the variation between adjacent micro-subdomains is linear, and as the spacing between micro-subdomains decreases, this linear assumption becomes more accurate. Figure 5b–d show how the streamwise density profiles for case S3 (40 micro-subdomains) compared to the profile measured by the full MD simulation at three snapshots in time. Our results show good agreement with the noisy MD data.

**Fig. 6:**
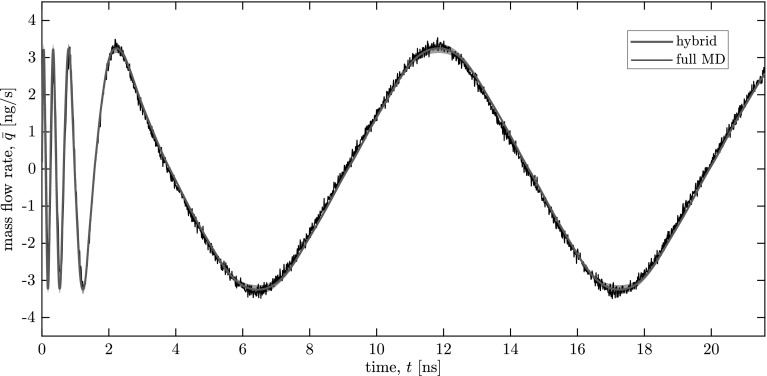
Transient mass flow rate results for our hybrid solution (blue line) and the full MD simulation (black line) for Case 2—micro-subdomain #1 (variable frequency external force). The hybrid solution uses an uncertainty threshold of 0.1 ng/s, and the initial database is that which was generated at the end of Case 1

Another example of building on an existing database is evaluating the response to different external forcing functions in the same geometry. Without the aid of a surrogate model, this would require performing an entirely new hybrid or full MD simulation. Using the database generated at the end of Case 1 as our initial database, we perform one new case: Case 2, with a variable-frequency external force whose oscillation period starts from 0.22 ns and gradually increases to 10.8 ns; the amplitude is 0.487 pN as in Case 1. Figure 6a shows the transient mass flow rate results for our hybrid solution and the full MD solution (Case D in Borg et al. 2015). Once again, our solution exhibits strong agreement with the full MD simulation, and the computational speed-up is effectively infinite^7^ as no new micro-simulations are performed.

### Generalisation and limitations

The on-the-fly GP regression approach presented here is applicable to a wide range of hybrid methods, with the capacity for more complex macro- and micro-models to be incorporated. Broadly speaking, this can present three challenges: 1) the micro-model passes more variables to the macro-model, e.g. some form of constitutive relation like viscosity or slip length; 2) the macro-model passes more variables to the micro-model, e.g. the flow rate is temperature dependent; or 3) the micro- and macro-models are more tightly coupled, such that the quasi-steady assumption is no longer true for the micro-simulations.

Challenge #1 is the most simple: you can just use a separate GP for each output variable. This will not particularly increase the computational cost of the surrogate model as each GP will be independent, so regression can be solved in parallel. In challenge #2, the dimensionality of input space for the surrogate model increases with each new variable. This means that exponentially more data points are required to map a sufficient quantity of input space for our surrogate to make accurate predictions. The main computational cost of regression is inverting the covariance matrix—used in Eqs. (6) and (7)—which is of a size $N_{\mathrm {sim}}\times N_{\mathrm {sim}}$, where we recall that $N_{\mathrm {sim}}$ is the number of micro-simulations performed (i.e. number of data points). However, this task does likely not become prohibitive until the number of data points is ${\mathcal {O}}(10000)$, and even then there are methods to perform regression using a subset of the covariance matrix $M_{\mathrm {sim}}$ (see Rasmussen and Williams 2006 Chapter 8 for details), reducing the cost from ${\mathcal {O}}(N_{\mathrm {sim}}^3)$ to ${\mathcal {O}}(N_{\mathrm {sim}}M_{\mathrm {sim}}^2)$.

Challenge #3 is the more conceptually difficult, but it eventually becomes another form of challenge #2. If we cannot perform steady-state micro-simulations because the macro- and micro-models are no longer scale-separated in time, then each micro-simulation requires more input variables to define its progress. For example, in our benchmark system perhaps the initial velocity profile over the channel may be required, along with the simulated time. The velocity profile would be binned over the channel height and so require several inputs. This does have the potential to make input space incredibly large, but if a shape could be assumed for the velocity profile then this is less of a problem. These challenges aside, the main problem GPs have is that they struggle to represent discontinuities, as Gaussians are smooth functions. However, as long as it is smooth, a GP is capable of modelling any function.

## Conclusion

We have presented an enhancement to conventional hybrid methods in fluid dynamics, using Gaussian process regression on the fly to predict microscopic detail based purely on macroscopic information, thereby avoiding costly repeated simulations of similar atomistic configurations. This procedure enables micro-information to be reused multiple times, drastically increasing the computational efficiency without adversely affecting the accuracy.

We compare our new scheme to full molecular dynamics (MD) simulations and find strong agreement, with errors within the range of thermal noise when a tight uncertainty threshold is set (up to 0.3 ng/s). As this threshold is raised, the error increases to over three$\times$ thermal noise (0.05 ng/s); however, the computational speed-up over a full MD simulation also increases. When starting from an empty database, raising the threshold from 0.1 to 0.9 ng/s increases speed-up from $12.3\times$ to $69.3\times$ with a resulting decrease in accuracy from 0.035 to 0.169 ng/s. Thus, the choice of threshold is a trade-off between the required accuracy and computational efficiency.

We demonstrate the computational benefit of creating an initial database to train our predictive model, by estimating the expected range of input values. This enables more predictions to be made via interpolation between known data, which provides less uncertainty than extrapolation and means fewer micro-simulations are performed ‘on-the-fly’. While maintaining approximately the same level of accuracy, starting with a modest initial database covering just 16 data points resulted in a speed-up of $30\times$ the full MD simulation for an incurred error of 0.042 ng/s.

Finally, we show how existing databases can be built upon (while never needing to be fully complete) to rapidly obtain high-resolution hybrid solutions—i.e. cheaply add more micro-subdomains at locations of interest—or to model different flow fields effectively instantly—i.e. no new micro-simulations are required.

## Appendix A. MD parameters

Non-equilibrium MD simulations of dense fluid argon are performed using mdFoam (Macpherson and Reese 2008; Borg et al. 2010)—an in-house solver developed in OpenFOAM. Atoms are modelled as hard spheres which interact using shifted Lennard-Jones (LJ) pair potentials, with wall atoms frozen in place. Our MD parameters are necessarily identical to those used by Borg et al. (2015) for the full MD simulations. The LJ potential

A.1 $$\begin{aligned} U_{LJ}(r_{ij}) = 4\epsilon \left[ \left( \frac{\sigma }{r_{ij}}\right) ^{12} - \left( \frac{\sigma }{r_{ij}}\right) ^{6}\right] , \end{aligned}$$

has a cut-off radius of 1.36 nm; for the fluid-fluid interactions, the LJ characteristic length and energy are $\sigma _{f-f}=0.34$ nm and $\epsilon _{f-f}=1.65678 \times 10^{-21}$ J, respectively; for the wall-fluid interactions, these parameters are $\sigma _{w-f}=0.255$ nm and $\epsilon _{w-f}=0.33 \times 10^{-21}$ J, respectively. The mass density of the wall atoms is $6.809 \times 10^{3}\, \hbox {kg}/{{\text{m}}^3}$, and the mass of a single atom is $m=6.6904 \times 10^{-26}$ kg. Fluid atom dynamics are described by Newton’s laws of motion, which are numerically integrated using the Velocity Verlet method (Swope et al. 1982), with a time-step of 5.4 fs. The excess heat generated by applying an external force is removed by modifying the velocities in the *z*-direction (see Fig. 1) using a Berendsen thermostat; this ensures a thermally homogeneous system, maintained at a temperature of 292.8 K. All of our simulations are periodic in the *s*- and *z*-directions. Mass flow rate and pressure measurements are time-averaged over 40000 time-steps.

## Appendix B. Exponential kernel $d^2_{ij}$ term

In the exponential kernel, the term $d^2_{ij}$ is the squared Euclidean distance between input points $\mathbf{x }_i = (h_i,\rho _i,F_i)$ and $\mathbf{x }_j = (h_j,\rho _j,F_j)$. For our benchmark system, we have chosen to normalise these distances by the mean separation distance for each input variable $(\Delta {\bar{h}},\Delta {\bar{\rho }},\Delta {\bar{F}})$. The mean separation distance is calculated using the points in our training database and its purpose is to simplify the surrogate model by reducing the number of hyperparameters, enabling the use of a single length scale $\ell$ instead of a separate length scale for each input variable. The equation for $d^2_{ij}$ is thus

B.1 $$\begin{aligned} d^2_{ij} = \left( \frac{h_i - h_j}{\Delta {\bar{h}}}\right) ^2 + \left( \frac{\rho _i - \rho _j}{\Delta {\bar{\rho }}}\right) ^2 + \left( \frac{F_i - F_j}{\Delta {\bar{F}}}\right) ^2. \end{aligned}$$

## Footnotes

1 Here, we use the notation that a lower case symbol is a scalar, a bold symbol is a vector, and a capitalised symbol is a matrix.

2 The magnitude of this variance is assumed to be independent of the input values—i.e. the function is homoscedastic. This is a reasonable assumption for isothermal flow of a dense fluid.

3 If there are multiple data points within close proximity of the test input, then the uncertainty can be lower than the measurement noise.

4 In micro-simulations with a channel height of $h_1$, the combination of the largest force (0.487 pN) and the lowest density ($1120\, \hbox {kg/m}^3$) produced a shear rate beyond the critical limit (Thompson and Troian 1997), so the mass flow rate does not converge. Therefore, one data entry is removed from all relevant databases.

5 $K(t_i,t_j) = \sigma _f^2\mathrm {exp}(-2\sin ^2(\pi \vert t_i-t_j\vert /T/\ell ^2)) + \delta _{ij}\sigma _n^2$, where $T=10.8$ ns is the oscillation period, $\sigma _f= 1$ ng/s, $\ell = 1$ s, and $\sigma _n = 0.1$ ng/s.

6 It is difficult to provide similar analysis for the density ranges, because at the nanoscale, viscosity varies rapidly with density, so greater density does not necessarily produce a larger mass flow rate.

7 Of course, the speed-up is not actually infinite, but the computational effort to run the macro-model is negligible compared to an micro-simulation.
